# Melatonin Prevents Chemical-Induced Haemopoietic Cell Death

**DOI:** 10.3390/ijms15046625

**Published:** 2014-04-17

**Authors:** Sara Salucci, Sabrina Burattini, Michela Battistelli, Valentina Baldassarri, Davide Curzi, Aurelio Valmori, Elisabetta Falcieri

**Affiliations:** 1 DiSTeVA, University of Urbino Carlo Bo, Urbino 61029, Italy; E-Mails: sabrina.burattini@uniurb.it (S.B.); michela.battistelli@uniurb.it (M.B.); valentina.baldassarri@uniurb.it (V.B.); davide.curzi@uniurb.it (D.C.); elisabetta.falcieri@uniurb.it (E.F.); 2 IGM, CNR, Rizzoli Orthopaedic Institute, Bologna 40136, Italy; E-Mail: valmori@area.bo.cnr.it

**Keywords:** chemical triggers, melatonin, apoptosis, U937

## Abstract

Melatonin (MEL), a methoxyindole synthesized by the pineal gland, is a powerful antioxidant in tissues as well as within cells, with a fundamental role in ameliorating homeostasis in a number of specific pathologies. It acts both as a direct radical scavenger and by stimulating production/activity of intracellular antioxidant enzymes. In this work, some chemical triggers, with different mechanisms of action, have been chosen to induce cell death in U937 hematopoietic cell line. Cells were pre-treated with 100 μM MEL and then exposed to hydrogen peroxide or staurosporine. Morphological analyses, TUNEL reaction and Orange/PI double staining have been used to recognize ultrastructural apoptotic patterns and to evaluate DNA behavior. Chemical damage and potential MEL anti-apoptotic effects were quantified by means of Tali^®^ Image-Based Cytometer, able to monitor cell viability and apoptotic events. After trigger exposure, chromatin condensation, micronuclei formation and DNA fragmentation have been observed, all suggesting apoptotic cell death. These events underwent a statistically significant decrease in samples pre-treated with MEL. After caspase inhibition and subsequent assessment of cell viability, we demonstrated that apoptosis occurs, at least in part, through the mitochondrial pathway and that MEL interacts at this level to rescue U937 cells from death.

## 1. Introduction

Melatonin (MEL) is a pineal hormone that is responsible for numerous functions. It regulates the sleep-wake cycle maintaining the circadian rhythms and it is used in pharmacological doses to resynchronize the internal biological clock. Furthermore, it modulates gonadal function, stimulates the immune response, has anti-inflammatory actions and exhibits strong antioxidant effects. This last MEL property lies in its powerful antioxidant functions [1–5], which are able to scavenge hydroxyl free radicals and many other related molecules. In various experimental models of tissue damage, its protective role was reported by reducing oxidative stress and lipid peroxidation [6,7]. The ability of MEL to reduce radical production, increasing the activity of many antioxidant enzymes [8], has been demonstrated in oxidant-related pathological conditions, where radical species are measured in extracellular fluids [9,10].

The mechanism through which MEL stimulates antioxidant enzymes is unclear: the increase of antioxidant defence is a cell response to oxidative stress, and the ability of an antioxidant such as MEL to promote cell defence remains an intriguing issue [8].

However, studies based on the activity of MEL on cell cultures give a completely different picture of its effect, describing an intense pro-oxidant activity of the hormone in certain conditions [11–13]. High concentrations of MEL (more than 100 μM) enhance the effect of chemotherapeutic drugs, both in cultured cancer cells [14–19] and *in vivo* [20,21], suggesting that in particular conditions MEL can be harmful, if not deleterious, for cancer cells.

Current evidences indicate that alterations of the intracellular redox state play a key role in the high concentration MEL effect against cancer cells, decreasing the cell proliferation rate and stimulating oxidative conditions which lead to apoptosis. The last data may be in conflict with the fact that high concentrations of MEL show also clear antioxidant properties [22,23].

In addition, cells can generate endogenous reactive oxygen species (ROS), useful in the induction and maintenance of several signal transduction pathways [24,25], which involve the activation of nuclear transcription factors to control the expression of genes related both to survival and death [26–30].

A survey of the literature shows that in leukocytes MEL mainly exerts an anti-apoptotic role [31–33], suggesting that it may support the viability of cells engaged in active/acute responses. In a recent study, MEL has been used at pharmacological concentrations to control mitochondrial damage and apoptotic signalling of UVB-irradiated leukemic cells. In the presence of the caspase-9 inhibitor ZVAD-Fmk, MEL seems to drive UVB-stressed cells to follow the intrinsic apoptotic pathway, interfering just at the mitochondrial level. Moreover, treatment with MEL or with ZVAD-Fmk prevent the K^+^ current reduction observed after UVB application, sparing cells from death [33].

Data in literature demonstrate MEL anti-apoptotic activity, both in normal and cancer cells, is due to its radical-scavenging properties [34]. Furthermore, in previous works carried out by our research group, it was shown that MEL prevents apoptosis induced by UVB radiation by interacting with the mitochondrial pathway [6,35]. In this work, MEL effect on the cytotoxicity induced by hydrogen peroxide (H_2_O_2_) and by staurosporine has been investigated by means of morphological, molecular and quantitative approaches in U937 cell line.

## 2. Results and Discussion

All analyses indicate that both treatments generate a diffuse cell damage and that MEL administration is able to reduce it. First of all, a viability test to evaluate which MEL concentration counteracts the chemical effect has been carried out.

**Figure 1.**
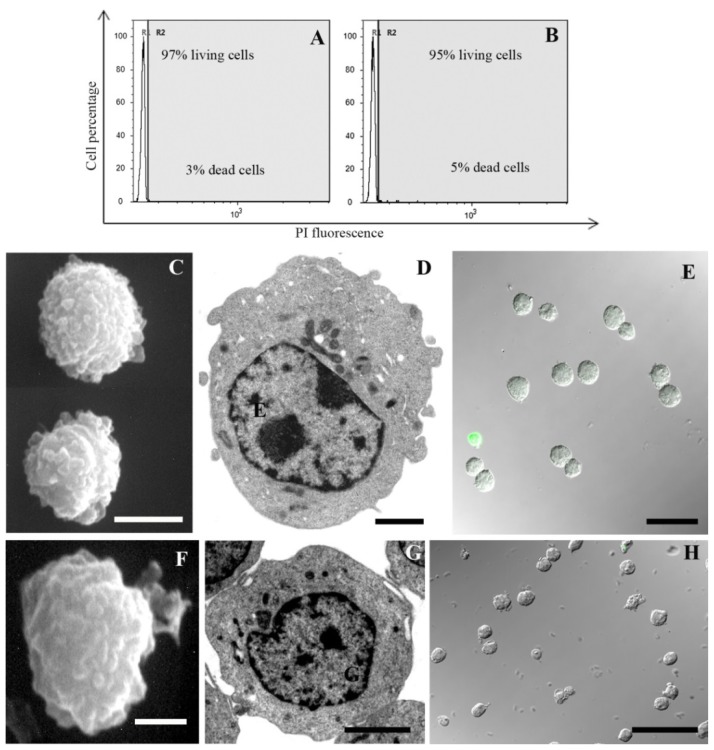
Control (**A**,**C**–**E**) and MEL alone (**B**,**F**–**H**) treated cells analysed by means of supravital PI (**A**,**B**), SEM (**C**,**F**), TEM (**D**,**G**) and CLSM (**E**,**H**) reveal a negligible cell death and normal morphological features. Histograms (**A**,**B**) show in white and grey area, living and dead cells, respectively. Scale bars: (**C**) 5 μm; (**D**,**F**,**G**) 2 μm; (**E**,**H**) 10 μm.

**Figure 2.**
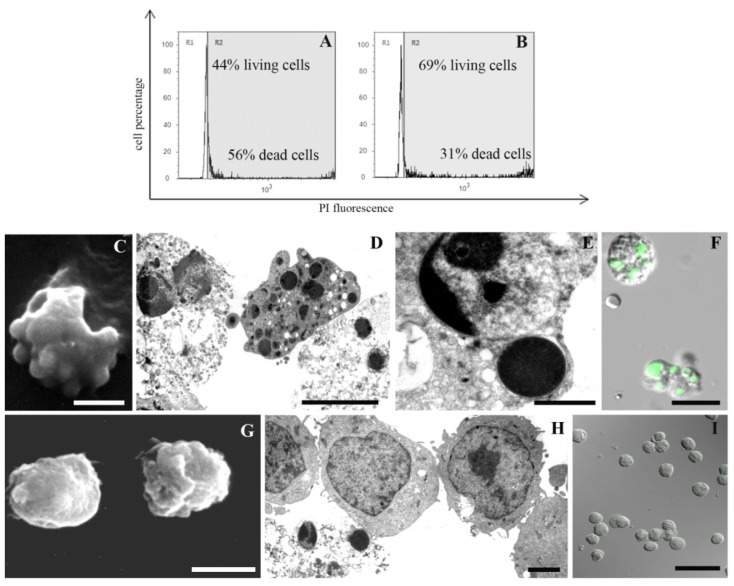
Cells exposed to H_2_O_2_ (**A**,**C**–**F**) or MEL+ H_2_O_2_ (**B**,**G**–**I**), analysed by means of supravital PI, SEM, TEM and CLSM, prove a cell viability decrease (**A**) in the presence of morphological apoptotic patterns: membrane blebbing (**C**), micronuclei (**D**,**E**) and necrotic cell death (**D**). In addition, several positive nuclei appear after TUNEL reaction (**F**). MEL administration is able to ameliorate cell viability (**B**) and to reduce apoptotic events (**G**–**I**). Scale bars: (**C**,**D**,**F**,**G**) 5 μm; (**E**,**H**) 2 μm; (**I**) 10 μm.

**Figure 3.**
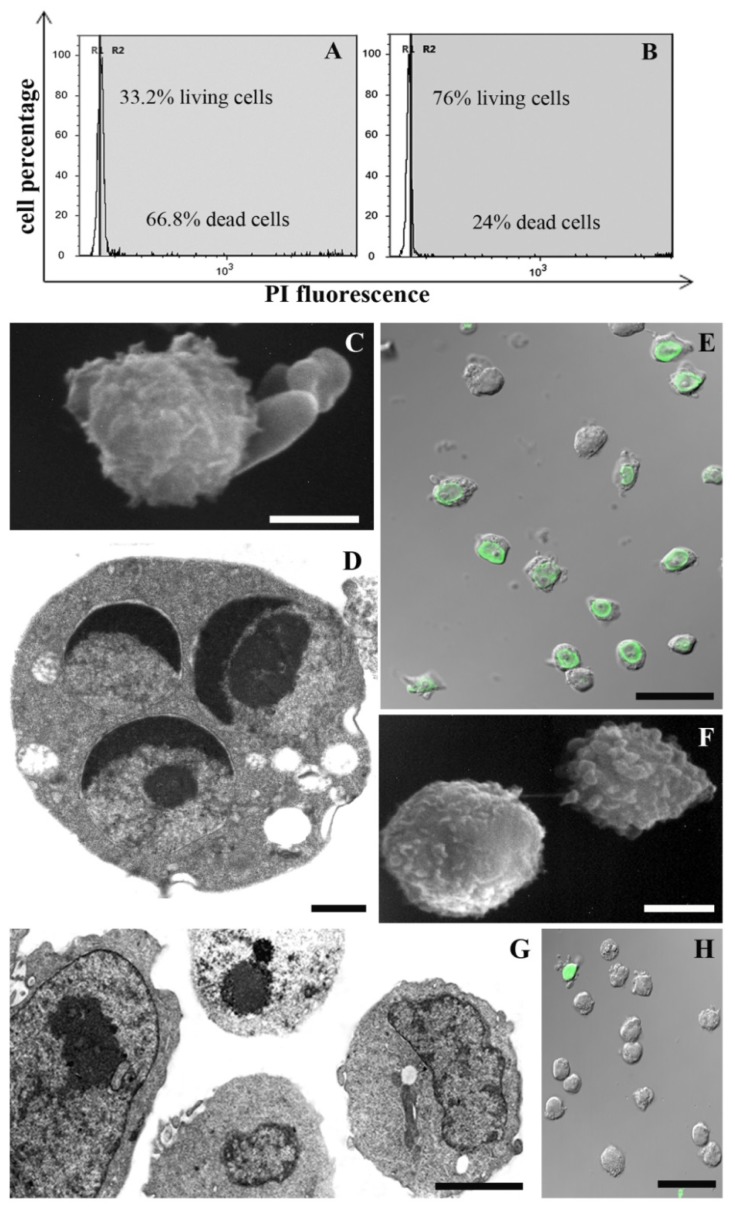
Cells exposed to staurosporine (**A**,**C**–**E**) or MEL + staurosporine (**B**,**F**–**H**), analysed by means of supravital PI (**A**,**B**), SEM (**C**,**F**), TEM (**D**,**G**) and CLSM (**E**,**H**). The trigger induces an evident cell death increase (**A**); apoptotic features can be observed after ultrastructural analyses (**C**–**E**). MEL added before staurosporine treatment restores cell viability (**B**) and morphology (**F**,**G**) and prevents DNA fragmentation *in situ* (**H**). Scale bars: (**C**,**F**) 5 μm; (**D**) 1 μm; (**E**,**H**) 10 μm; (**G**) 2 μm.

A 1 mM MEL dose employed with success against UVB-induced cell death [6,35] had a scant effect against chemical damage (data not shown). When decreasing its concentration to 100 μM, an improvement of cell viability appears. In particular, supravital PI analysis revealed a preserved cellular condition in control samples (Figure 1A; 97% living cells) and in cells exposed to MEL alone (Figure 1B; 95% living cells). Cellular viability decreased after H_2_O_2_ (Figure 2A; 44% living cells) or staurosporine (Figure 3A; 33% living cells) exposure. MEL pre-treatment significantly reduced cell death (Figures 2B and 3B; 69% and 76% living cells, respectively).

SEM and TEM showed a good cell viability in control condition (Figure 1C–E) and in cells treated with MEL alone (Figure 1F–H). After H_2_O_2_ and staurosporine exposure, a certain cell death rate could be detected at morphological analyses and it decreased in MEL pre-treated samples. Membrane blebbing appeared at SEM after chemical treatments (Figures 2C and 3C) while it was absent after MEL pre-incubation before chemical stress (Figures 2G and 3G). At TEM, cells exposed to H_2_O_2_ evidenced both apoptotic and necrotic patterns (Figure 2D,E). Several apoptotic cells appeared after staurosporine treatment (Figure 3D). In all conditions, chromatin condensation, in addition to micronuclei scattered throughout the cytoplasm and a diffuse cytoplasmic vacuolization have been observed. When cells were pre-treated with MEL, before cell death induction, a better morphology was observed in all experimental conditions with an evident attenuation of apoptotic patterns (Figures 2G and 3H).

MEL anti-apoptotic effect has been confirmed also with the TUNEL reaction. Fluorescent nuclei were absent in the control sample (Figure 1E) and in MEL alone (Figure 1H) treated cells. After H_2_O_2_ (Figure 2F) and staurosporine (Figure 3E) exposure, several TUNEL positive nuclei appeared, numerically decreasing when MEL was added before H_2_O_2_ (Figure 2I) or staurosporine (Figure 3H) administration.

**Figure 4.**
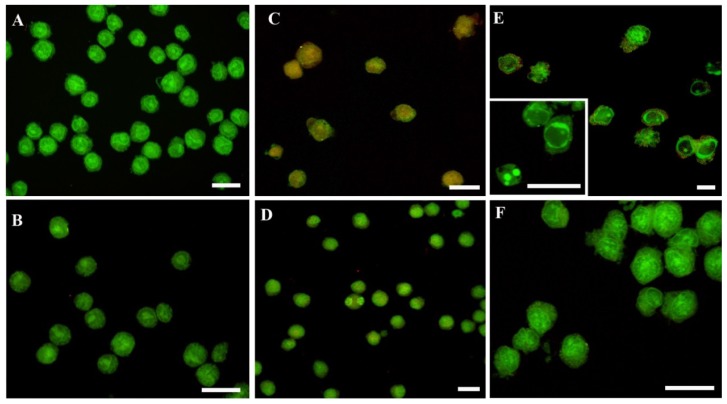
Fluorescent micrographs of AO and PI double-stained U937 cells. Intact green nuclei appear in control (**A**) and MEL alone (**B**) conditions suggesting a good cell viability. After H_2_O_2_ (**C**) or staurosporine (**E** and inset) exposure apoptotic cells appear and their decrease can be observed in MEL pre-treated samples (**D**,**F**). Scale bars: (**A**–**F**) 25 μm; (**E** and inset) 10 μm.

In addition, CLSM evaluation of living, apoptotic and dead cells after AO/PI double staining (Figure 4) showed the same behavior of MEL in preventing cell death. In control (Figure 4A) and MEL alone (Figure 4B) conditions, cells showed preserved green nuclei, indicating a good viability. H_2_O_2_ exposure induced late apoptotic and necrotic stages (orange fluorescence, Figure 4C) while staurosporine (Figure 4E) is characterized by early apoptotic features with chromatin margination in cup-shaped masses and micronuclei presence (bright green fluorescence, inset E). These apoptotic patterns decreased in MEL-treated samples (Figure 4D,F), where cells showed a morphology and a green fluorescence similar to control and MEL alone conditions.

To quantify MEL ability in preventing apoptosis, U937 cells were analysed with Annexin V and PI, able to recognize both early and late apoptotic cells as well as necrotic cell population. Moreover, caspase-9 inhibitor was added to cell medium before chemical exposure and the effect was analysed through this apoptotic protocol, to highlight mitochondrial pathway involvement.

**Figure 5.**
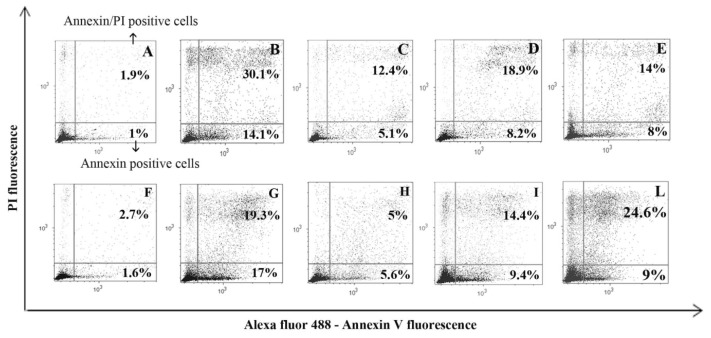
Annexin V/PI dot plots in control condition (**A**), MEL alone (**F**), H_2_O_2_ (**B**) and staurosporine (**G**)-treated cells. In (**C**) and (**H**), dot plots are relative to MEL administration before H_2_O_2_ and staurosporine respectively. Dot plots of caspase-9, −3 inhibitor pre-treatment before apoptosis induction appear in (**D**–**E)** and (**I**–**L**).

In Figure 5, a dot plot for each experimental condition has been reported as a representative experiment. Dot plot showed a negligible apoptotic cell number in control and MEL alone specimens (Figure 5A,F) and the capacity of used triggers to induce a certain apoptotic and necrotic rate (Figure 5B,G) which decreased in samples pre-treated with MEL (Figure 5C,H). If the pre-incubation with caspase-9 inhibitor prevented, at least in part, cell death (Figure 5D,I), a different behavior appeared when cells were pre-treated with the caspase-3 inhibitor before apoptosis induction. Caspase-3 inhibition showed a living cell number increase in H_2_O_2_ treatment, suggesting the caspase-dependent pathway involvement for this trigger (Figure 5E). On the other hand, in the case of staurosporine-treated cells after caspase-3 inhibition, Annexin/PI positive cells increased (Figure 5L).

**Figure 6.**
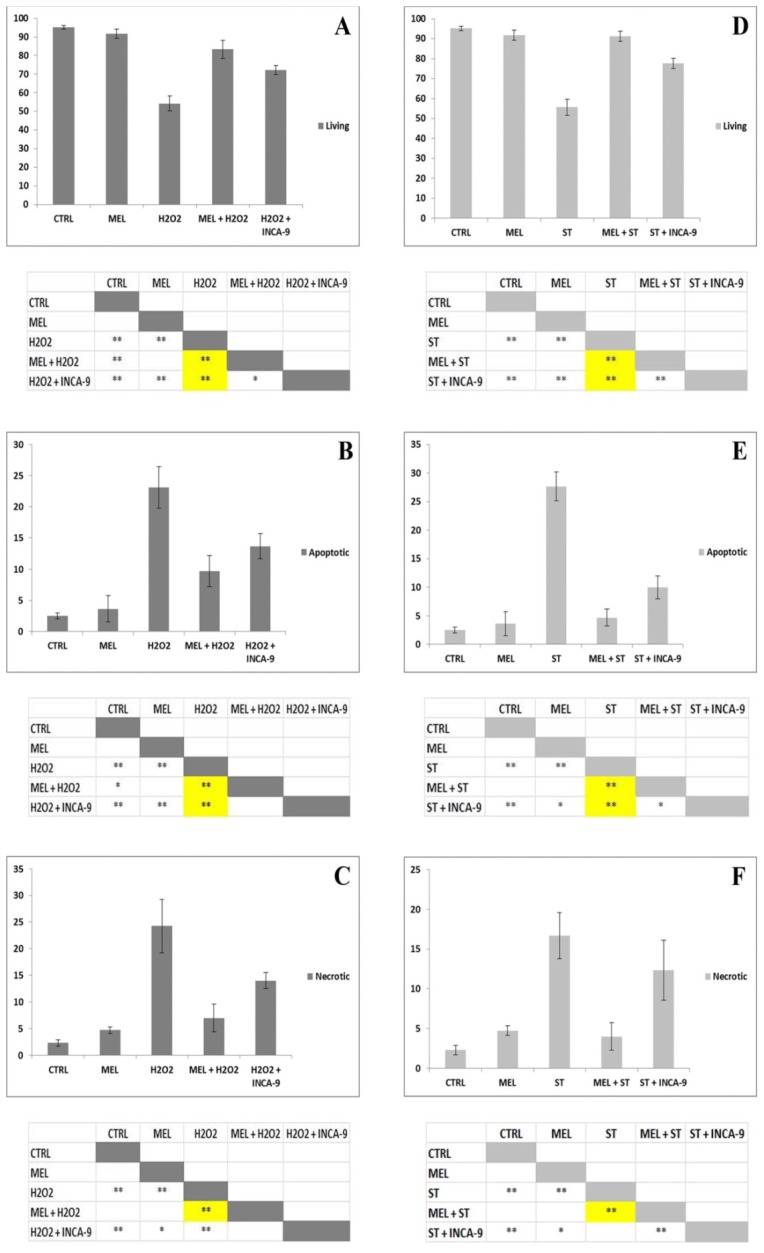
Percentage study of viable, apoptotic and necrotic cells based on Annexin V analyses. All data are expressed as mean values of percentages for each group ± DS. MEL significantly (yellow in the table) restore cell viability (**A**,**D**) and prevents apoptotic (**B**,**E**) and necrotic cell death (**C**,**F**), induced by H_2_O_2_ (**A**,**B**,**C**) or staurosporine (**D**,**E**,**F**) with caspase-9 involvement.

The histograms (Figure 6), corresponding to dot plots, showed that all triggers significantly reduced cell viability, if compared to the control condition and MEL alone treatment (Figure 6A,D). Both triggers induced apoptotic (Figure 5B,E) and, in a lesser extent, necrotic cell death (Figure 6C,F). MEL administration before H_2_O_2_ or staurosporine exposure was able to significantly prevent both apoptosis and necrosis (Figure 6B–F). Moreover, in the presence of caspase-9 inhibitor, living cell number (Figure 6A,D) significantly increased if compared to the correspondent chemical treatment, suggesting the involvement of the intrinsic pathway in apoptosis induction. The apoptotic cell number statistically decreased in caspase-9 inhibitor pre-treated samples (Figure 6B,E).

## 3. Experimental Section

### 3.1. Cell Culture

Human myelomonocytic lymphoma cell line U937 was grown in RPMI 1640, supplemented with 10% heat-inactivated fetal bovine serum, 2 mm glutamine, 1% antibiotics and was maintained at 37 °C in humidified air with 5% CO_2_.

For the induction of apoptosis, U937 cells (seeded at 1 × 10^6^ cells/mL) were exposed to 0.5 mM H_2_O_2_ or 0.5 μM staurosporine for 4 or 5 h, respectively. These chemicals have been chosen on the basis of their known apoptotic effect and of their different mechanisms of action, which are able to increase ROS levels [36,37]. Cells were pre-treated with 100 μM MEL for 24 h before apoptosis induction. MEL (Sigma, St. Louis, MO, USA) was first dissolved in absolute ethanol at the initial concentration of 100 mM, and then diluted at final 100 μM concentration in culture medium. Cell behavior was monitored by means of the inverted microscopy.

### 3.2. Tali^®^ Image-Based Cytometer

The Tali^®^ Image-Based Cytometer (Life Technologies Europe BV, Monza, Italy) is a 3-channel (bright field, green fluorescence, red fluorescence) benchtop assay platform that offers several advantages over flow cytometry and fluorescence microscopy.

It is capable of performing a range of suspension cell-based assays, including green fluorescent protein (GFP) and red fluorescent protein (RFP) expression, apoptosis and cell viability assays. It captures up to 20 images (fields of view) per sample, automatically analyses the images with sophisticated digital image-based cell counting and fluorescence detection algorithms [38].

#### 3.2.1. Supravital Propidium Iodide (PI; Tali^®^ Viability Kit; Life Technologies)

A 100 μL aliquot of cells (1 × 10^6^) from each sample was treated directly in cell culture medium with 1 μg/mL PI [39] for a few minutes and then analysed by means of Tali^®^ Image-Based Cytometer, able to recognize and count living and dead cells. Dot plots have been elaborated with the Attune Cytometric Software.

#### 3.2.2. Annexin V and PI (Tali^®^ Apoptosis Kit; Life Technologies)

Apoptosis can be distinguished from necrosis by characteristic morphological and biochemical changes, including compaction and fragmentation of the nuclear chromatin, shrinkage of the cytoplasm, and loss of membrane asymmetry. In normal living cells, phosphatidylserine is located on the cytoplasmic surface of the cell membrane. In apoptotic cells, however, it is translocated from the inner to the outer membrane. Alexa Fluor^®^ 488—Annexin V allows the detection of phosphatidylserine exposed on the outer cell membrane following caspase-activation, in combination with nuclear PI staining [35]. A 100 μL aliquot of cells (1 × 10^6^) from each sample was centrifuged and resuspended in 100 μL apoptosis buffer added to 5 μL Annexin V (conjugated whit Alexa Fluor^®^ 488) and incubated at room temperature in the dark for 20 min. Then, samples were centrifuged and resuspended again in 100 μL of the same buffer and added with 1 μL PI at room temperature in the dark for 1–4 min and analysed at Tali^®^ Image-Based Cytometer. For each analysis, dot plot and real percentages have been elaborated with the Attune Cytometric software. Alexa Fluor^®^ 488—Annexin V and PI were set on the logarithmic scale for all experiments.

#### 3.2.3. Caspase-9 and −3 Inhibitor Evaluation with Annexin V and PI

Each sample was pre-treated for 2 h with 5 μL caspase-9 (Ac-LEHD-CMK, a specific caspase-9 inhibitor, Calbiochem, Billerica, MS, USA) or caspase-3 inhibitor (Ac-DMQD-CHO, a selective irreversible caspase-3 inhibitor; Calbiochem) before H_2_O_2_ or staurosporine exposure and its relationship with apoptosis has been investigated by means of Tali^®^ Apoptosis Kit (Annexin V Alexa Fluor^®^ 488 and PI; Life Technologies) following the experimental protocol described above.

#### 3.2.4. Statistical Procedures

Differences in the percentages of viable, apoptotic, and necrotic cells among groups were determined using one-way analysis of variance (ANOVA) followed by Tukey HSD *post hoc* tests to evaluate individual group differences. Significance was set at *p* < 0.05. Data were collected from three independent experiments.

ANOVA analysis. Living cells (A). Single factor ANOVA: F(4,10) = 75.33 *p* < 0.01; ** Tukey HSD *p* < 0.01 H_2_O_2_ *vs.* CTRL, MEL, MEL + H_2_O_2_ and H_2_O_2_ + INCA-9; MEL + H_2_O_2_ *vs.* CTRL; H_2_O_2_ + INCA-9 *vs.* CTRL and MEL; * Tukey HSD *p* < 0.05 MEL + H_2_O_2_ *vs.* H_2_O_2_ + INCA-9. Apoptotic cells (B). Single factor ANOVA: F(4,10) = 39.87 *p* < 0.01; ** Tukey HSD *p* < 0.01 H_2_O_2_ *vs.* CTRL, MEL, MEL + H_2_O_2_ and H_2_O_2_ + INCA-9; H_2_O_2_ + INCA-9 *vs.* CTRL and MEL; * Tukey HSD *p* < 0.05 MEL + H_2_O_2_ *vs.* CTRL. Necrotic cells (C). Single factor ANOVA: F(4,10) = 32.18 *p* < 0.01; ** Tukey HSD *p* < 0.01 H_2_O_2_ *vs.* CTRL, MEL, MEL + H_2_O_2_ and H_2_O_2_ + INCA-9; H_2_O_2_ + INCA-9 *vs.* CTRL; * Tukey HSD *p* < 0.05 H_2_O_2_ + INCA-9 *vs.* MEL. Living cells (D). Single factor ANOVA: F(4,10) = 109.72 *p* < 0.01; ** Tukey HSD *p* < 0.01 ST *vs.* CTRL, MEL, MEL + ST and ST + INCA-9; ST + INCA-9 *vs.* CTRL, MEL and MEL + ST. Apoptotic cells (E). Single factor ANOVA: F(4,10) = 94.26 *p* < 0.01; ** Tukey HSD *p* < 0.01 ST *vs.* CTRL, MEL, MEL + ST and ST + INCA-9; ST + INCA-9 *vs.* CTRL; * Tukey HSD *p* < 0.05 ST + INCA-9 *vs.* MEL and MEL + ST. Necrotic cells (F). Single factor ANOVA: F(4,10) = 21.7 *p* < 0.01; ** Tukey HSD *p* < 0.01 ST *vs.* CTRL, MEL and MEL + ST; ST + INCA-9 *vs.* CTRL and MEL + ST; * Tukey HSD *p* < 0.05 MEL *vs.* ST + INCA-9.

### 3.3. Scanning Electron Microscopy (SEM)

U937 cells were cultured and treated in flask and, after washing in 0.1 M phosphate buffer, they were fixed in suspension with 2.5% glutaraldehyde in the same buffer for 1 h. Afterwards, they were deposited on poly-l-lysine-coated coverslips overnight at 4 °C. All the specimens were post-fixed with 1% OsO_4_ in 0.1 M phosphate buffer for 1 h. After alcohol dehydration, they were critical point dried, gold sputtered and observed with a Philips 515 scanning electron microscope (UMKC, Kansas City, MO, USA) [40].

### 3.4. Transmission Electron Microscopy (TEM)

Differently treated U937 pellets were immediately fixed in 2.5% glutaraldehyde in 0.1 M in phosphate buffer (pH 7.3). The cells were then post-fixed in 1% OsO_4_ in the same buffer, dehydrated with ethanol and embedded in araldite as previously described [41]. For ultrastructural analysis, thin sections were collected on nickel grids, stained with uranyl acetate and lead citrate, and observed with a CM10 electron microscope (FEI Italia SRL, Milano, Italy).

### 3.5. TUNEL

Control and treated cells were fixed with 4% paraformaldehyde in phosphate buffer saline (PBS) pH 7.4 for 30 min. They were then deposited on poly-lysinated coverslips in Petri dishes overnight at 4 °C. All samples were rinsed with PBS and permeabilized with a 2:1 mixture of ethanol and acetic acid for 5 min at −20 °C. For the TUNEL technique, all reagents were part of the Apoptag Plus kit (D.B.A., Oncor, Dallas, TX, USA) and procedures were carried out according to the manufacturer’s instructions. Cells were treated with TdT buffer for 10 min at room temperature and incubated with the reaction buffer containing the TdT enzyme, for 1 h at 37 °C in a humidified chamber. The reaction was blocked using the stop buffer for 10 min. Cells were incubated with a FITC-conjugated anti-digoxigenin antibody for 30 min at room temperature. Finally, slides were mounted with an antifading medium [42]. Specimens were observed with a Leica TCS-SP5 confocal laser scanning microscope (CLSM) connected to a DMI 6000 CS inverted microscope (Leica Microsystems CMS GmbH, Mannheim, Germany); excitation was at 488 nm and emission signals were detected at 517 nm. CLSM Images are presented as maximum intensity projection or single-plane images.

### 3.6. Acridine Orange (AO) and PI Nuclei Staining

Cells, fixed with 4% paraformaldehyde in PBS pH 7.4 for 30 min and deposited on poly-lysinated coverslips in Petri dishes, were washed twice using PBS. Cells were pre-treated with RNasi A 10 μg/mL in PBS for 30 min and then exposed to an equal mixture of PI (1 μg/mL; Life Technologies) and AO (1 μg/mL; Life Technologies) diluted in PBS at room temperature in the dark for 10 min.

Specimens were observed with a Leica TCS-SP5 CLSM connected to a DMI 6000 CS Inverted Microscope (Leica Microsystems CMS GmbH; FICT and PI excitation were at 488 and 500 nm, respectively, and their emission signals were detected at 617 and 525.) CLSM Images are presented as maximum intensity projection or single-plane images.

AO and PI are intercalating nucleic acid specific fluorochromes which emit green and red fluorescences, respectively, when they are bound to DNA. Only AO can diffuse through the plasma membrane of viable and early apoptotic cells. Viable cells show green nucleus with intact structure while apoptotic cells exhibit a bright-green nucleus showing condensation of chromatin in dense green areas. Late apoptotic and necrotic cells are stained with both AO and PI. Comparatively, PI produces the highest intensity emission in necrotic cells. Hence, late apoptotic cells exhibit an orange nucleus showing condensation of chromatin, whereas necrotic cells display a red nucleus [43].

## 4. Conclusions

This study emphasizes MEL role as an anti-apoptotic molecule. In our model, MEL pre-treatment significantly inhibits H_2_O_2_-induced apoptosis—thanks to its capacity, thoroughly described by several authors [5,6]—to protect against ROS. It also significantly prevents apoptosis by exposing cells to staurosporine, a molecule that has a dual mechanism of action *i.e.*, ROS increase and p53-dependent apoptosis.

These findings, obtained through different experimental techniques, demonstrate the MEL ability in preventing apoptotic cell death induced by chemical triggers.

In all conditions, apoptotic morphological features—such as chromatin condensation, cup-shaped masses, micronuclei formation, apoptotic body production and cells in secondary necrosis—have been observed and their disappearance has been revealed in samples pre-treated with MEL. Moreover, these chemicals are able to induce a DNA cleavage *in situ*, as identified by means of the TUNEL technique. After MEL administration, an evident nuclear staining decrease appeared, suggesting a lack of DNA fragmentation.

The anti-apoptotic ability of MEL has been demonstrated by evaluating phosphatidylserine exposure, another typical apoptotic feature, by means of Annexin V/PI staining. MEL administration before chemicals significantly increases cell viability. Moreover, the use of caspase-9 inhibitor demonstrates that for both considered chemicals apoptosis is accomplished through the intrinsic apoptotic pathway and that MEL could act, at least in part, at this level to exert its protection. Moreover, the analyses of executive caspase-3 shows a different behavior of two triggers in inducing cell death. In fact, the caspase-3 inhibitor evaluation increases living cell number after H_2_O_2_ exposure, suggesting for this trigger a caspase-dependent involvement to induce apoptosis. After caspase-3 inhibitor administration, before staurosporine exposure, a moderate annexin V/PI positive cell increase has been observed, suggesting for this chemical agent a different behavior. The intrinsic pathway, activated by staurosporine treatment, evidences that mitochondria are the primary targets which can release apoptotic proteins that stimulate different downstream effectors from the apaf–1/caspase 9 complex such as AIF and other molecules. Some studies have demonstrated that staurosporine can activate an additional pathway that is not inhibited by anti-apoptotic Bcl-2 proteins, but mediated by an apaf-1-independent activation of caspase-9 [44], suggesting the presence of a caspase-independent signalling [45], even if the death-receptor one cannot be excluded.

Thus, MEL is able to counteract chemical apoptotic cell death, probably by interacting with the mitochondrial target. These findings are in agreement with our previous works reporting that MEL prevented UVB cell death by stabilizing the mitochondrial membrane potential and by inhibiting cytochrome c release [6,35]. Many of the beneficial effects of MEL administration may depend on its action on mitochondria [46–48] where high concentrations of MEL were found. In fact, there is evidence that mitochondria may have the capacity to synthesize and metabolize MEL [49,50]. It has been demonstrated that MEL pharmacological doses affected the mitochondrial function and prevented mitochondrial dysfunction under pathological conditions [51–53]. In addition, a recent paper [54] reported that MEL ability in reducing cell damage seems due to its capacity in decreasing oxidative stress and in stimulating GHS level.

The mechanism through which this occurs remains unclear. However, some evidences suggest MEL capacity to regulate the Bcl-2 family activity [55], which may be an alternative way to explain its antioxidant role [34,56,57]. Thus, mitochondria may be considered the principle site where MEL exerts its anti-apoptotic action [35,58]. These data are in agreement with our results that demonstrated MEL capacity to modulate mitochondrial pathway in preventing apoptotic chemical damage.

These findings confirm that MEL can be recognized, in particular experimental conditions, as an anti-apoptotic tool, which protects human lymphoma U937 cells from apoptosis induced by H_2_O_2_ and staurosporine.
